# Tyrosine-specific MAPK phosphatases and the control of ERK signaling in PC12 cells

**DOI:** 10.1186/1750-2187-1-4

**Published:** 2006-11-29

**Authors:** Yvet E Noordman, Patrick AM Jansen, Wiljan JAJ Hendriks

**Affiliations:** 1 Department of Cell Biology, Nijmegen Centre for Molecular Life Sciences, Radboud University Nijmegen Medical Centre, Geert Grooteplein 28, 6525 GA Nijmegen, The Netherlands

## Abstract

**Background:**

Spatio-temporal control of extracellular signal-regulated kinase (ERK) activity, a critical determinant of the cell's response to growth factors, requires timely dephosphorylation of its regulatory tyrosine and/or threonine residue by MAPK phosphatases. We studied the physiological role of kinase interaction motif (KIM)-containing protein tyrosine phosphatases (PTPs) in the control of EGF- and NGF-induced ERK activity in neuroendocrine PC12 cells.

**Results:**

We found a single KIM-containing PTP to be endogenously expressed in rat PC12 cells: the transmembrane PTPRR isoform termed PCPTP1. Protein knock-down of PCPTP1, or fourfold overexpression of its mouse orthologue, PTPBR7, left EGF- and NGF-induced ERK1/2 activity in PC12 cells unaltered. Ectopic expression of cytosolic PTPRR isoforms, however, resulted in reduced EGF-induced ERK1/2 activity, an effect that was dependent on the phosphatase activity and the KIM-domain of these PTPs.

**Conclusion:**

The finding that robust changes in tyrosine-specific MAPK phosphatase expression levels have minor effects on temporal ERK1/2 activity control in PC12 cells suggests that dual-specificity MAPK phosphatases may act as major regulators of growth factor-induced ERK1/2 signaling in these cells.

## Background

One of the best-studied cellular signaling relays is the mitogen-activated protein kinase (MAPK) signaling cascade, the central mechanism by which growth factors steer cellular key decisions such as proliferation and differentiation [1]. It has become clear that it is not just the amplitude but also the spatio-temporal distribution of the MAPK activity within the cell that determines the final outcome of this signaling relay. For example, in the rat PC12 phaeochromocytoma cell line seminal studies have shown that epidermal growth factor (EGF)-induced proliferation relies on a transient activation of the extracellular signal-regulated kinases 1 and 2 (ERK1/2) in the cytosol, whereas nerve growth factor (NGF)-induced differentiation requires a sustained ERK1/2 activation and translocation to the nucleus [2,3]. Thus, not only signaling molecules that activate these ubiquitous MAPKs but also the ones that inactivate and/or localize them are important for determining signaling specificity. ERK activation requires the phosphorylation of two closely spaced regulatory tyrosine and threonine residues by the dual specificity MAP kinase kinases MEK1/2 [4]. Concomitantly, ERK inactivation involves dephosphorylation of these residues by MAPK phosphatases. Although the EGF and NGF specific pathways that lead to ERK activation have been mapped in detail in PC12 cells [3], the contribution of MAPK phosphatases in this model system has received little attention.

The dephosphorylation of ERK1/2 regulatory tyrosine and threonine residues can be achieved by three different phosphatase types: protein serine/threonine phosphatases, dual specificity (serine/threonine/tyrosine) phosphatases (MKPs/DUSPs) and classical phosphotyrosine-specific protein tyrosine phosphatases (PTPs) [5]. The latter two types have gained considerable interest because they contain a so-called kinase interaction motif (KIM) that is also present in the upstream MAPK kinases MEK1/2. The KIM-domain mediates the interaction and in part determines binding specificity towards the different MAPKs [6-8]. Importantly, this interaction can be regulated via cAMP because PKA-mediated phosphorylation of a serine residue within the KIM-domain abolishes binding and dephosphorylation of MAPKs [9,10].

**Figure 1:**
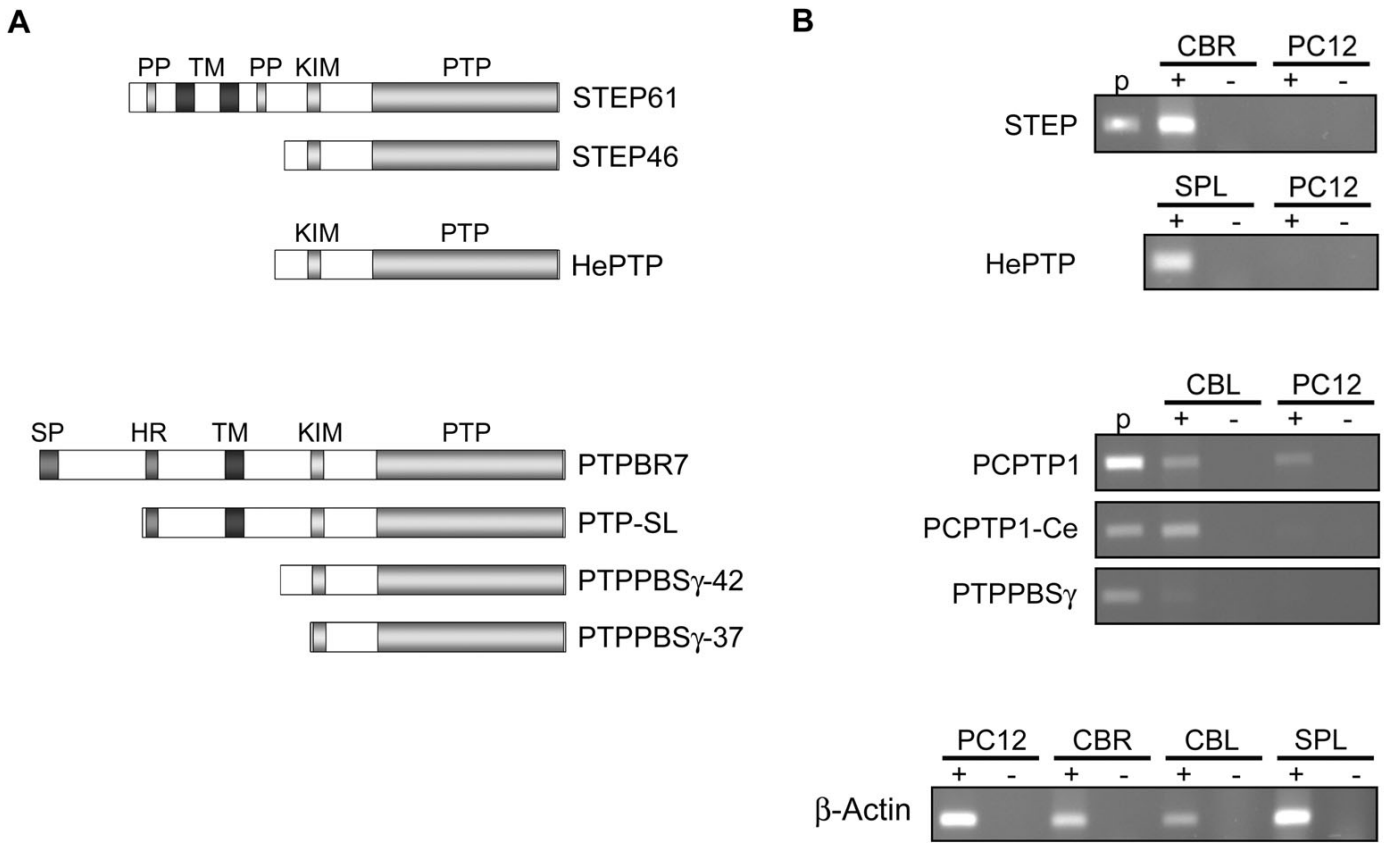
**Tyrosine-specific MAPK phosphatases expressed in PC12 cells**. A) Schematic diagrams of STEP (STEP61, STEP46), HePTP and PTPRR (PTPBR7, PTP-SL, PTPPBSγ-42, PTPPBSγ-37) protein isoforms. Mouse isoform nomenclature is given on the right. Signal peptides (SP), proline-rich and PEST domains (PP), transmembrane segments (TM), kinase-interacting motifs (KIM), hydrophobic regions (HR) and catalytic phosphotyrosine phosphatase domains (PTP) are indicated. B) Assessment of KIM-containing, tyrosine-specific PTP expression by RT-PCR. Rat protein nomenclature is indicated on the left, aligned with the diagrams of the mouse orthologs depicted in panel A). STEP and HePTP transcripts, as detected by RT-PCR (+) in mouse cerebrum (CRB) and spleen (SPL) RNA, respectively, are not found in PC12 cells. For rat PTPRR proteins (PCPTP1, PCPTP1-Ce, PTPPBSγ) isoform-specific primer sets were used on rat cerebellum (CBL) and PC12 total RNA. Samples lacking RT enzyme (-) and appropriate plasmids (p) served as negative and positive controls, respectively. A β-actin specific RT-PCR served as a positive control for the reverse transcriptase reactions.

Mammals contain three classical PTP families that may regulate ERK activity by KIM-dependent binding and dephosphorylation: HePTP/LC-PTP, STEP and PTPRR (Fig. 1A). The haematopoietic HePTP/LC-PTP was indeed shown, through gene targeting studies, to be a physiological regulator of ERK in lymphocytes [11] and the striatal-enriched phosphatase (STEP) enzyme family was found to regulate ERK activity in primary neuronal cultures [12]. For PTPRR isoforms current data mainly reflect overexpression studies in non-neuronal cells [6,9,13]. Given that rat PTPRR receptor-type isoform (PCPTP1) mRNA levels in PC12 cells are increased nine-fold within 8 h following NGF treatment [14], a role in neuronal differentiation induced by sustained ERK activity may be expected.

In the current study we assessed the contribution of the KIM-domain-containing classical PTPs to the regulation of ERK activity in rat PC12 cells. HePTP and STEP mRNAs were not detectable in PC12 cells. For the PTPRR gene a single transcript type, encoding the transmembrane isoform PCPTP1, was encountered. We exploited retroviral transduction to increase or decrease PTPRR protein levels and monitored growth factor-induced ERK activation using phosphospecific antibodies. Our results demonstrate that KIM-domain-containing tyrosine-specific PTPs are dispensable for proper ERK-dependent signaling in PC12 cells and put forward the dual-specificity MAPK phosphatases as the physiological regulators of ERK activity in these cells.

## Results

### A single classical KIM-containing PTP is expressed in PC12 cells

To determine which of the three KIM-containing phosphotyrosine-specific PTPs are endogenously expressed in rat PC12 cells, an RT-PCR analysis on total RNA was performed. Expected amplicons for STEP and HePTP were obtained from mouse cerebrum and mouse spleen RNA, respectively, but STEP and HePTP messengers remained undetectable in PC12 material (Fig. 1B), in concordance with earlier reports [15,16]. For the rat PTPRR gene two different transcripts, encoding PCPTP1 and PCPTP1-Ce, respectively, have been reported [17,18]. PCPTP1 mRNA is present in PC12 cells [14,17,19] but PCPTP1-Ce transcripts remain undetectable in PC12 RNA, although the amplification product is readily obtained using rat cerebellum RNA (Fig. 1B). In mouse the PTPRR-encoding gene generates a third transcript isoform which encodes two additional protein isoforms (PTPPBSγ-42 and PTPPBSγ-37) through the use of alternative translation start sites [20]. To be able to probe for the presence of putative rat PTPPBSγ variants, we searched in public databases for the rat PTPRR genomic region displaying very high homology to mouse PTPPBSγ transcript-specific sequences and designed primers for use in RT-PCR assays. Indeed, the predicted PTPPBSγ-type fragment was amplified from rat cerebellum RNA, while mouse PTPPBSγ cDNA served as positive control, but no signal was obtained using PC12 material (Fig. 1B). Thus, in rat PC12 cells only the largest PTPRR transcript, that encodes the receptor-type isoform PCPTP1, is expressed.

**Figure 2:**
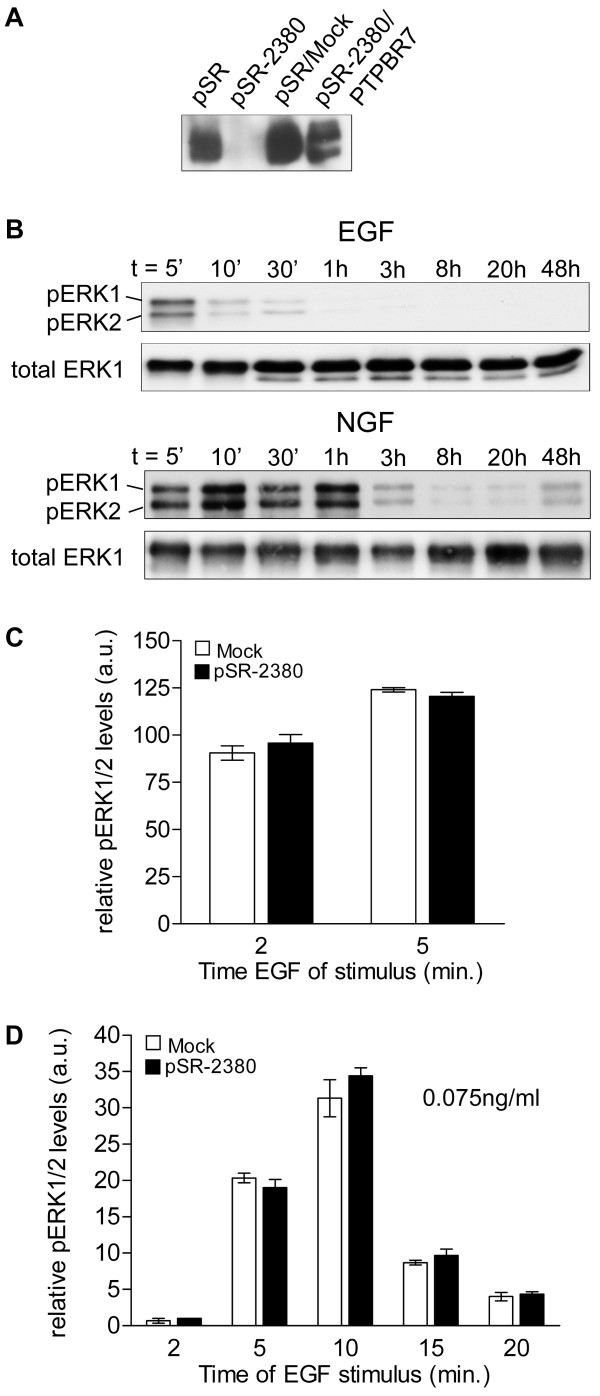
**ERK activity profile is not affected by PCPTP1 depletion**. A) RNAi-mediated knockdown of endogenous PCPTP1 in PC12 cells. PC12 cells stably expressing siRNA targeted to nucleotides 2380–2398 in PCPTP1 cDNA (pSR-2380) were generated via retroviral transduction. Empty vector (pSR) was used as control and PCPTP1 knockdown was rescued by transduction of pSR-2380-containing cells with a retroviral expression construct for the mouse ortholog PTPBR7. Equal amounts of protein from the resulting stable cell pools were subjected to immunoprecipitation using α-SL antiserum and captured proteins were analyzed on Western blots using monoclonal antibody 6A6. B) Transient and sustained ERK1/2 signaling in wild type PC12 cells. After overnight serum deprivation, cells were stimulated with 100 ng/ml EGF (upper panels) or 50 ng/ml NGF (lower panels) for the indicated time points. Cell lysates were subjected to Western blot analysis and phospho-ERK1/2 signals (pERK1, pERK2) were detected using a chemiluminescence imaging system. The same blot was reprobed with total ERK1 antibody to correct for loading differences. C) Transient ERK activity is not affected in PCPTP1 knockdown cells. Serum-starved cells were stimulated with 10 ng/ml EGF for 2 or 5 min, respectively, and harvested. Relative phospho-ERK1/2 levels in the protein lysates, determined as above, are presented as mean values ± SEM from three independent experiments. D) PCPTP1 knockdown in PC12 cells does not significantly alter ERK activity at reduced levels of EGF. Serum starved cells were stimulated with 0.075 ng/ml EGF for the indicated time points and relative phospho-ERK1/2 levels were determined as before. Results are mean values ± SEM (n = 4).

We next investigated PTPRR isoform expression in PC12 cells at the protein level. Both Western blot analysis of PC12 cell lysates and immunoprecipitation experiments revealed multiple immunoreactive bands (e.g. Fig. 2A and data not shown) that compare well to patterns obtained with expression constructs for rat PCPTP1 [17]. Proteins of around 72 and 65 kDa in size are detected that represent endogenous rat PCPTP1 and a post-translationally modified variant, respectively, as is the case for the mouse ortholog PTPBR7 [20]. These data are in line with PTPRR isoform PCPTP1 to be the only detectable KIM-containing phosphotyrosine-specific phosphatase in PC12 cells.

### RNAi-mediated knock-down of PCPTP1

To assess whether dephosphorylation by PCPTP1 is of relevance for the regulation of EGF- and NGF-induced ERK signaling in PC12 cells, we aimed at silencing PCPTP1 expression via RNA interference. Different siRNA sequences were tested through viral transduction with pSUPER-Retro [21] and the one targeted to nucleotides 2380–2398 of PCPTP1 resulted in an almost complete ablation of PCPTP1 protein levels (Fig. 2A). To enable exclusion of off-target effects in these cells, knockdown was also rescued by introduction of a retroviral expression vector encoding PTPBR7, the mouse ortholog of PCPTP1. Effects on ERK activity following EGF or NGF administration to serum-starved PC12 cells were monitored over time by immunoblot analysis of cell lysates using phosphospecific ERK1/2 antibodies. Reprobing the same immunoblot with antiserum directed against total ERK1 enabled us to correct for loading differences between samples and to express ERK1/2 activity as relative phosphorylation units. EGF administration to mock-transduced PC12 cells resulted in a very rapid but transient ERK1/2 activation that peaks within the first 5 min (Fig. 2B, upper panels). Upon NGF treatment, ERK1/2 biphosphorylation is maximal after some 10 min and then gradually decreases, with sustained ERK1/2 activity still being detectable after 48 h (Fig. 2B, lower panels) [22]. Reasoning that possible effects of the altered PCPTP1 levels would be most noticeable at time points with maximal ERK1/2 activation, we compared relative phospho-ERK1/2 levels in the transduced cells at 2 and 5 min after EGF addition (Fig. 2C) or at 10, 20, 30 and 60 min following NGF administration (data not shown). Depletion of PCPTP1, however, did not result in significant alteration of the response to EGF or NGF administration. Also the use of very low EGF concentrations, to ensure that ERK1/2 is not maximally phosphorylated and PCPTP1 knockdown may allow further increase of ERK1/2 activity, did not reveal a significant difference in ERK1/2 activation between mock transduced and PCPTP1 knockdown cells (Fig. 2D).

### Effects of PTPRR overexpression on transient and sustained ERK activity

**Figure 3:**
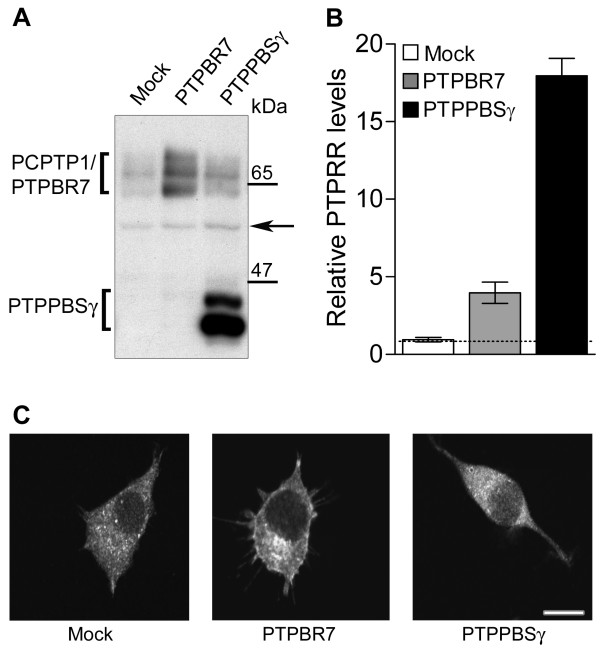
**Expression of PTPRR isoforms in PC12 cells**. A) PC12 cells were retrovirally transduced with expression constructs for mouse PTPRR isoforms, or mock-transduced. Equal amounts of cell lysates were subjected to 10% SDS-PAGE and immunoblotting. Rat PCPTP1, mouse PTPBR7 and mouse PTPPBSγ (indicated on the left) were detected using 6A6 antibody. Molecular size markers are indicated on the right. The arrow indicates a cross-reactive ~50 kDa background band. B) Quantitative representation of PTPRR expression levels as determined in A. PTPRR levels were corrected for loading differences using monoclonal α-tubulin antibody E7 immunostaining (not shown) as a control. Results are the mean values ± SEM of four individual pools of cells. C) Subcellular localization of stably expressed PTPRR protein isoforms in the transduced PC12 cells as determined by fluorescence microscopy using polyclonal α-SL antibody. Bar indicates 10 μm.

As an alternative way to assess the relevance of PTPRR activity for the regulation of ERK signaling, we generated pools of PC12 cells that stably expressed mouse PTPRR isoforms by means of retroviral transduction. Lysates of the resulting cell lines were first analyzed on Western blots to determine PTPRR expression levels (Fig. 3A). Transduction with mouse PTPBR7-encoding viruses resulted in immunoreactive bands that superimposed on those of endogenous PCPTP1, confirming the previous annotation of these signals. Ectopic expression of mouse PTPPBSγ transcripts resulted in two additional immunoreactive bands which represent PTPPBSγ-42 and PTPPBSγ-37 [20]. Cells transduced with a construct encoding PTP-SL, the mouse ortholog of PCPTP1-Ce, failed to display appreciable levels of expression (not shown) and are therefore not included in this study. Quantitation revealed that PTPBR7 expression exceeds three-fold the endogenous PCPTP1 level, whereas PTPPBSγ expressing cells show PTPRR levels that are some seventeen-fold higher than in non-transduced controls (Fig. 3B). Analysis of the subcellular distribution revealed that endogenous PCPTP1 displayed a weak vesicle-type of staining in PC12 cells, and only limited cell membrane localization (Fig 3C, left panel). In line with previous data [20,23,24], its mouse ortholog PTPBR7 was detected at the cell membrane, on vesicular structures and at the Golgi apparatus (Fig. 3C, middle panel). Mouse PTPPBSγ isoforms are cytosolic proteins that are excluded from the nucleus, while endogenous PCPTP1 adds a speckled pattern (Fig. 3C, right panel).

**Figure 4:**
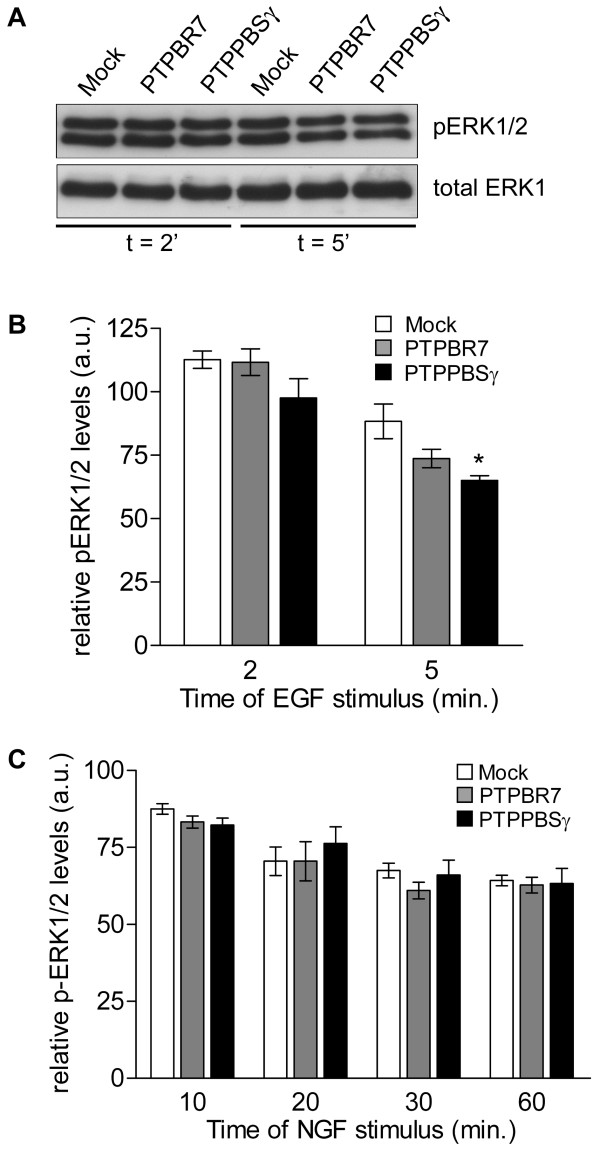
**PTPRR effect on growth factor-induced ERK signaling**. A) PC12 cells stably expressing PTPPBSγ show reduced transient ERK1/2 activity. Serum-starved pools of mock-transduced, PTPBR7 expressing or PTPPBSγ expressing cells were treated with 100 ng/ml EGF for 2 or 5 min before being lysed. Proteins were size-separated and immunoblotted. Representative images of phospho-ERK1/2 (upper panel) and total ERK1 (lower panel) immunoreactivity are depicted. B) Reduced transient ERK activity in PTPPBSγ expressing cells. Quantitative representation of relative phospho-ERK1/2 levels in cells stimulated with EGF for two or five minutes. Results are presented as mean values ± SEM from six independent experiments (Student's t-test, *p < 0.008). C) Increased PTPRR levels have no effect on NGF-induced ERK1/2 activity. Serum starved PC12 cell pools stably expressing PTPBR7 or PTPPBSγ were stimulated with 50 ng/ml NGF for the indicated time points (in min). Cells were then lysed and phospho-ERK1/2 were analysed on Western blots as in panels A-B. Results are the mean ± SEM of three independent experiments.

Effects of PTPBR7 and PTPPBSγ expression on ERK1/2 activity profiles in the transduced cells were again determined through phospho-ERK1/2 immunoblots (Fig. 4A). Mean values for six independent experiments were plotted (Fig. 4B,C) and identical results were obtained using independently derived PC12 stably transduced cell pools. Ectopic expression of PTPBR7 was found to have no effect on EGF-induced transient and NGF-induced sustained ERK1/2 activity. At 5 min following EGF addition the PTPBR7 expressing cells display a small reduction in ERK1/2 activity, but this does not reach significance. Likewise, at 2 min following EGF administration and at 10, 20, 30 or 60 min after NGF treatment the phospho-ERK1/2 levels in the various cell pools expressing PTPPBSγ are indifferent from those in mock-transduced cells. On the contrary, PTPPBSγ expressing cells show a significant reduction in ERK1/2 activity levels 5 min after EGF administration (p < 0.008; Fig. 4B). Separate analyses of the ERK1 and ERK2 activity levels provided essentially the same results (data not shown). Thus, boosting PTPRR activity in the cytosol 17-fold results in a significant ~25% reduction of ERK1/2 activity during the later phase of the transient response to EGF.

### PTPRR effects on ERK activity involve KIM-mediated interactions

**Figure 5:**
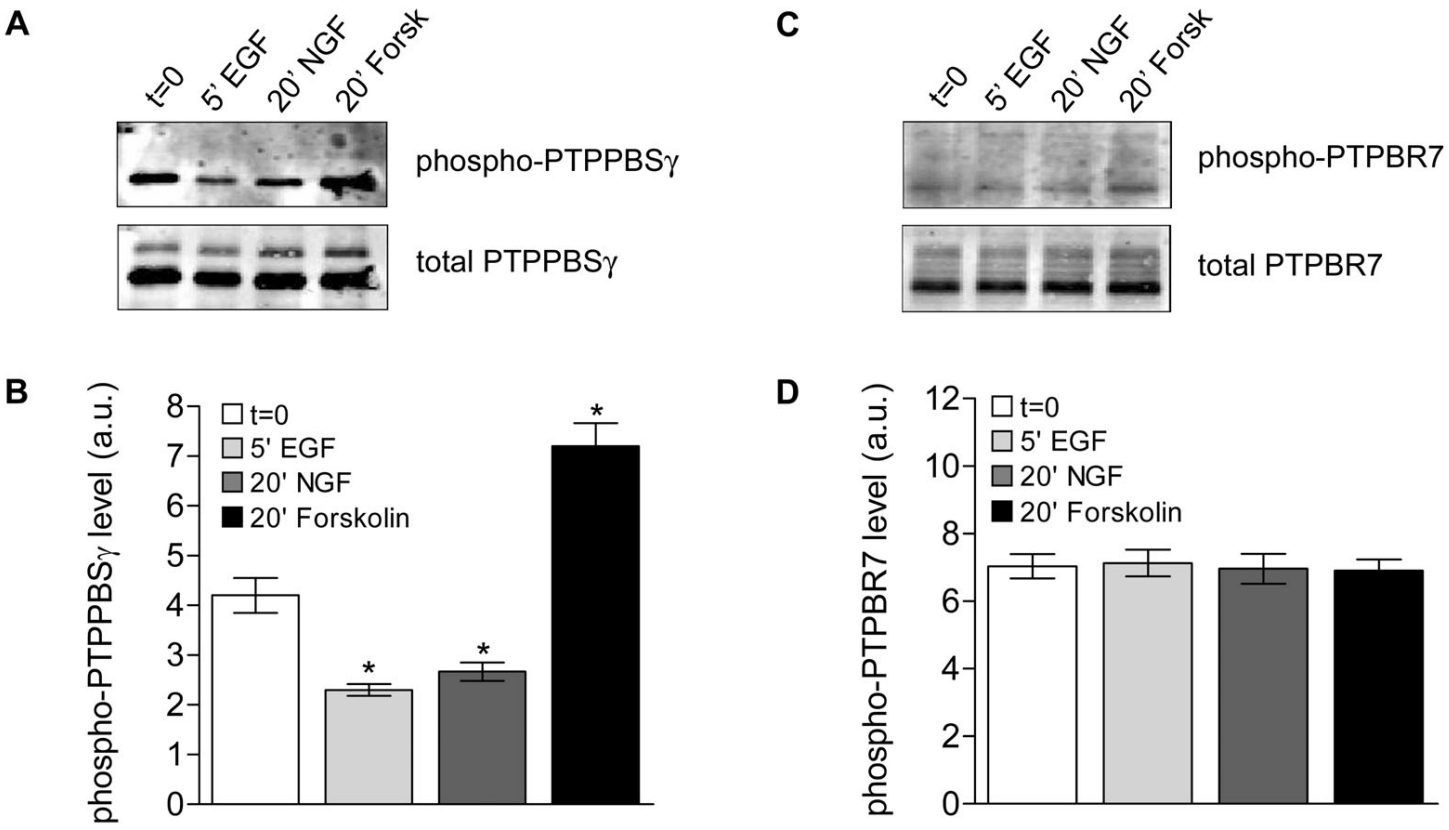
**Assessment of PTPRR KIM domain phosphorylation**. A) Lysates of PTPPBSγ expressing cells after overnight serum starvation (t = 0) and subsequent treatment with EGF, NGF or forskolin were subjected to immunoprecipitation with monoclonal antibody 6A6, and captured proteins were analyzed on blot using a phospho-PKA substrate antibody (upper panel) and 6A6 (lower panel). It was ascertained that the phospho-PKA substrate antibody remained unreactive towards PTPPBSγ mutant S94A (data not shown). B) Quantitative representation of phospho-PTPPBSγ levels determined as in A. Results, expressed as the ratio of the signals obtained with the phospho-PKA substrate antibody and the PTPRR antibody, are presented as mean values ± SEM (n = 3, Student's t-test, *p < 0.02). C) Representative image of PTPBR7 proteins, immunoprecipitated with antibody 6A6 from PTPBR7 expressing cells that received the indicated stimuli, as detected with phospho-PKA substrate antibody (upper panel) or 6A6 (lower panel). D) Quantitative representation of phospho-PTPBR7 levels as determined in C. Results are presented as mean values ± SEM (n = 3).

The binding of KIM-domain-containing phosphatases to their MAPK targets can be abrogated by the cAMP-dependent protein kinase PKA, through phosphorylation of a specific serine residue within the KIM-domain [9,10,12]. Intriguingly, current models on transient versus sustained ERK activation in PC12 cells incorporate an NGF-induced PKA-mediated signaling pathway [22]. To investigate whether this mechanism kept PTPRR isoforms from regulating ERK phosphorylation levels in PC12 cells, we investigated the phosphorylation status of the KIM domain serine residue in immunoprecipitated PTPRR proteins using an antibody directed against phosphorylated PKA substrates. Administration of the PKA activator forskolin was used to determine maximum levels of KIM-domain phosphorylation [12]. Phosphorylation levels of endogenous PCPTP1 in PC12 cells were, unfortunately, below detection level (data not shown). In PTPPBSγ-expressing PC12 cells about half of this phosphatase appeared phosphorylated following overnight serum starvation, and EGF or NGF administration even led to a significant decrease in the phosphorylation of the regulatory serine (Fig. 5A,B). In PTPBR7 expressing cells, however, the PTPBR7 KIM-domain seems maximally phosphorylated under all conditions tested (Fig. 5C, D), which may explain the negative findings regarding ERK1/2 activity modulation by this receptor-type PTPRR isoform.

**Figure 6:**
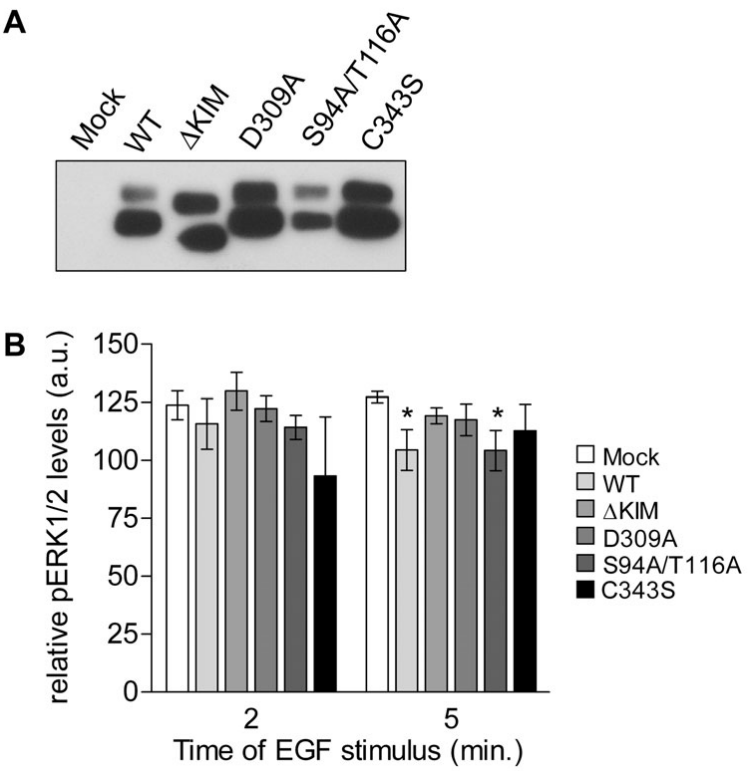
**Wild type and non-phosphorylatable PTPPBSγ affect ERK activity**. A) Expression analysis of wild type and mutant PTPPBSγ proteins. Equal amounts of protein from PC12 cells stably expressing wild type (WT) or mutant (ΔKIM, D309A, S94A/T114A, and C343S) PTPPBSγ were subjected to SDS-PAGE and immunoblot analysis using antibody 6A6. B) EGF-induced ERK1/2 activity in PC12 cells stably expressing PTPPBSγ protein variants. Serum starved cells that stably express PTPPBSγ mutants were stimulated with 10 ng/ml EGF for 2 or 5 min, and lysed. Relative phospho-ERK1/2 levels were determined via immunoblot analysis as described in the legends to Fig. 2. Results are presented as mean values ± SEM (n = 4, Student's t-test; *p < 0.05).

To determine whether the phosphatase activity and/or MAPK binding potential of PTPPBSγ is required for the observed attenuation of the EGF-stimulated transient ERK activity (Fig. 4), PC12 cell pools were generated that stably express specific PTPPBSγ mutants. PTPPBSγ C343S and D309A mutants represent catalytically impaired versions that still may bind phosphotyrosine-containing substrates [25]. In the ΔKIM mutant the KIM-domain has been deleted, making it unable to associate with MAPKs [6]. Furthermore, because phosphorylation is known to regulate its association with MAPKs [9,13], we also included the PTPPBSγ S94A/T116A double mutant in this series of experiments. The PTPPBSγ mutant-expressing cell pools demonstrated expression levels that were comparable with or even exceeding that of wild-type PTPPBSγ expressing cells (Fig. 6A). Again, relative phospho-ERK1/2 levels were determined by phospho-immunoblot analyses. Only in PC12 cells expressing wild type PTPPBSγ or PKA-insensitive PTPPBSγ S94A/T116A a significant decrease in ERK1/2 activity at 5 min, but not at 2 min, following EGF stimulation is apparent (Fig. 6B; p < 0.05). The catalytically impaired mutants as well as the version without the KIM-domain have lost this ability, demonstrating the requirement of a direct association with and subsequent dephosphorylation of the MAPK by the PTP.

## Discussion

The neuroendocrine PC12 cell line represents a paradigm model system to study spatial and temporal aspects of growth factor-induced MAPK signaling [2,26]. In this study we made an inventory of the MAPK-associating, phosphotyrosine-specific PTPs that are endogenously expressed in this cell system and tested their physiological relevance for the spatio-temporal control of EGF- and NGF-induced ERK1/2 activity. The PTPRR isoform PCPTP1 was the only detectable KIM-domain containing classic PTP in these cells. Knockdown of PCPTP1 protein levels or additional expression of its mouse ortholog PTPBR7, however, left transient and sustained ERK1/2 signaling in response to EGF or NGF, respectively, unaltered. Our finding that in this cell system PTPBR7 was constitutively phosphorylated on a regulatory serine residue within the KIM-domain, precluding its binding to ERK, provides a likely explanation. Ectopic expression of the cytosolic PTPPBSγ isoforms resulted in a decrease of EGF-induced, but not NGF-induced, ERK1/2 activation. This effect required PTPPBSγ enzymatic activity and an intact KIM-domain, pointing to a direct association with and dephosphorylation of the MAPK. In line with this, only some 30–50% of the PTPPBSγ molecules was found to be phosphorylated on their KIM serine residue, leaving the majority available for engagement in ERK binding and dephosphorylation.

It is unclear what causes the differential effect by the PTPPBSγ isoforms as compared to the receptor-type PTPBR7 protein on the amplitude of the transient ERK activity following EGF administration. It may well be attributable to the distinct subcellular localizations of these PTPRR isoforms or rather could reflect the much higher expression levels for PTPPBSγ-42/PTPPBSγ-37 as compared to PTPBR7. Why PTPPBSγ ectopic expression affects only the transient and not the sustained ERK activity profile, however, must have other grounds. Current models incorporate an NGF-induced PKA-mediated signaling pathway in PC12 cells [22] and, reasoning along these lines, PTPPBSγ proteins may thus become hyperphosphorylated in the KIM-domain upon NGF addition. In contrast, we found that treatment with EGF and NGF both resulted in significantly lower PTPPBSγ phosphorylation levels. This may be explained by assuming that the initial boost of ERK1/2 activity following growth factor addition results in a rapid KIM region-mediated association of PTPPBSγ and ERK1/2, thereby protecting the regulatory serine in the KIM region from subsequent phosphorylation. Irrespective, the PKA-mediated signaling that results from NGF treatment of PC12 cells, and that is required for the sustained nature of the ERK activity as compared to the transient EGF effects [22], does not explain the PTPPBSγ selective effect.

In line with our findings, ectopic overexpression of a cytosolic version of PTP-SL (thus resembling PTPPBSγ isoforms) in PC12 cells has been reported previously to result in reduced phospho-ERK5 and phospho-ERK1/2 levels following EGF administration [27]. Those data, however, were not quantified nor tested for statistical significance. In contrast with our studies, Ogata and coworkers did observe a suppressive effect of PTPBR7 on sustained ERK activity in PC12 cells [19]. Those experiments, however, involved transient transfections that likely result in much higher PTPBR7 expression levels, and activation of the PC12 cells was done through co-transfection of constitutively active MEK1 instead of NGF administration. Furthermore, not ERK phosphorylation status but transcriptional activity of its downstream substrate Elk1 was used as a read-out, and together this may have caused an exaggeration of effects.

PCPTP1, the rat homologue of mouse PTPBR7, is abundantly expressed in several rat brain regions, including the cerebellum, cerebral cortex and the hippocampus [17]. Intriguingly, PCPTP1 mRNA levels are increased 9-fold over the initial 8 h of NGF-induced differentiation of PC12 cells [14], in line with a role in neuronal differentiation induced by sustained ERK activity. Our protein knockdown experiment, however, are at odds with a critical role for PCPTP1 in ERK1/2-dependent processes in PC12 cells. Other KIM-domain-containing tyrosine-specific PTPs cannot camouflage the impact of PCPTP1 signaling since we excluded expression of other PTPRR isoforms, STEP and HePTP/LC-PTP in PC12 cells. On the other hand, redundancy with KIM- domain-containing dual-specificity phosphatases (MKP/DUSP) may well explain PTPRR's modest effect on ERK1/2 signaling. In *Drosophila*, for example, redundancy regarding ERK inactivation was encountered for DMKP3, a homolog of mammalian MKP-3/MKP-4, and PTP-ER, the *Drosophila *homolog of PTPRR [28]. Potential candidates that could take over the role of the PTPRR proteins in PC12 cells are MKP-3, MKP-4, MKP-X and Dusp2, in view of their localization in the cytoplasm and high specificity towards ERK1/2 [5]. We detected MKP-3 and MKP-X transcripts in PC12 cells by RT-PCR analyses (data not shown) but did not observe alterations in growth factor-induced ERK1/2 activity profiles following RNA interference strategies towards depletion of MKP-3 and MKP-X (data not shown). It should be noted that verification of changes in MKP protein levels was not performed due to lack of suitable antibodies.

What other substrate targets for PCPTP1 may exist in PC12 cells to explain its endogenous presence? Besides ERK1/2, PTPRR proteins were shown to bind the MAPK family members p38α and ERK5 [6,9,13,27,29] but these studies were mostly performed in non-neuronal cells, using either recombinant proteins or ectopically expressed phosphatases and MAPKs. Further studies towards the identification of the physiological targets of PTPRR isoforms are therefore needed. In addition, current *in vitro *studies might be hampered by the fact that potential ligands for the PCPTP1/PTPBR7 extracellular part, which may critically control the activity of the phosphatase, have not been identified yet. Also in view of the restricted expression pattern of the *Ptprr *gene [17,24,30] and its conserved potency to generate multiple protein isoforms through the use of different promoters, alternative splicing and multiple start codons [18,20,30] it may well be that the tyrosine phosphatase PTPRR family members will prove physiological regulators of MAPK signaling in other cell systems and following different stimuli than the ones tested here. Given the lack of endogenous PTPRR expression in many neuronal cell lines, studies in PTPRR deficient mice are eagerly awaited to address their functional significance in MAPK signaling *in vivo*.

## Conclusion

The PTPRR isoform PCPTP1 is the sole tyrosine-specific PTP in PC12 cells that is able to bind ERK MAPKs through its KIM-domain and it is readily up-regulated upon growth factor-induced differentiation of these cells. Still, the physiological regulation of ERK1/2 signaling in PC12 cells could not be ascribed to this PTP. We conclude that other, dual-specificity MAPK phosphatases may act as major regulators of EGF- and NGF-induced signaling pathways in these cells.

## Methods

### RT-PCR

Total RNA from exponentially growing PC12 cells, rat cerebellum, mouse cerebrum or spleen was purified using RNazol B (Campro Scientific, Veenendaal, The Netherlands). RT-PCR analyses were performed essentially as described [31]. Briefly, cDNA was synthesized from total RNA by random hexamer priming using SuperScript™ II RNase H^- ^Reverse Transcriptase (Invitrogen Life Technologies, Breda, The Netherlands) and used for PCR. Amplified products were analyzed on agarose gels. Primers used are: PCPTP1-forward 5'-CCTCAATGCACACACTATGAGG-3'; PCPTP1-Ce-forward 5'-CTTCAGCTCGCAGGC TTTC-3'; PTPPBSγ *(rat)*-forward 5'-GTGCAGGACGTGGAGGAAG-3'; PTPRR-reverse 5'-TCCTTCTTTGCTCCAGAT-3'; STEP-forward 5'-GAGGACTACCGGCTGCGAC-3'; STEP-reverse 5'-GGCTCATGGCGTGGTGCAC-3'; HePTP-forward 5'-CATCTGCTCAGT GAACACACC-3'; HePTP-reverse 5'-GTCTGCTACAGTGTTGGGC-3'; β-actin-forward 5'-GCTAGAGCTGCCTGACGG-3' and β-actin-reverse 5'-GAGGCCAGGATGGAGCC-3'.

### Plasmids

Subcloning of a *Xho*I-*Not*I fragment from pIRES2-eGFP (Clontech) into *Xho*I-*Not*I digested retroviral plasmid pLXSN (Clontech) generated pLXSN-IRES2-eGFP. The *Bam*HIsite in pLXSN-IRES2-eGFP was subsequently used to insert the *Bgl*II insert from pSG5-PTPPBSγ-FL [20], rendering pLXSN-PTPPBSγ-IRES2-eGFP. By inserting the N-terminus-encoding *Eco*RI fragment from PTPBR7-eGFP [23] into *Eco*RI-digested pLXSN-PTPPBSγ-IRES2-eGFP we generated pLXSN-PTPBR7-IRES2-eGFP. Mutant pLXSN-PTPPBSγ constructs were generated as follows. First mutant PTP-SL open reading frames were excised from pRK5-PTP-SL expression vectors ([6] kindly provided by dr. R. Pulido, Valencia, Spain) using *Not*I and *Xho*I, and ligated in between the corresponding sites in pLXSN. Using *Bst*BI we subsequently excised the mutation-containing sequence parts from the obtained plasmids and inserted them into *Bst*BI-digested pLXSN-PTPPBSγ-IRES2-eGFP. For siRNA constructs, appropriate oligonucleotides were annealed and ligated in the pSUPER-Retro vector pSR [21]. Plasmids pSR-1211 and pSR-2380 encode siRNAs that target the nucleotides 1211–1229 and 2380–2398 in the PCPTP1 mRNA [Genbank:D38292], respectively. All constructs were verified by sequence analysis.

### Cell culture and retroviral transduction

PC12 cells (ATCC #CRL-1721) were grown on collagen-coated dishes in DMEM supplemented with 10% FCS and 5% horse serum (HS) at 37°C. Ecotropic Phoenix packaging cells [32] were cultured in DMEM, 10% FCS. For retrovirus production, Phoenix cells were transiently transfected by calciumphosphate precipitation. Twenty-four hours after transfection, medium was replaced by DMEM containing 10% FCS and 5% HS. After 10 h retrovirus-containing medium was collected, filtered through a 0.45-μm pore-size filter and supplemented with polybrene (Sigma-Aldrich Chemie B.V., Zwijndrecht, The Netherlands) to a final concentration of 5 μg/ml. PC12 cells were infected by replacing culture medium with undiluted polybrene- and retrovirus-containing medium for 8–16 h. After four subsequent rounds of infection, transduced cells were selected for 2–3 weeks in medium containing 500 μg/ml G418 (Gibco Europe, Breda, The Netherlands) or 2.5 μg/ml puromycin (Sigma-Aldrich Chemie B.V.), respectively.

### Immunoblot analysis

Cells were seeded in six-well plates at a density of 1*10^6 ^cells/well. The next day medium was replaced by DMEM without serum. After o/n serum deprivation, cells were treated with 0.075–100 ng/ml EGF (a kind gift of Dr. J. van Zoelen, Nijmegen, The Netherlands) or 50 ng/ml NGF (N6009, Sigma-Aldrich Chemie B.V.) for different time periods. Cells were washed twice with ice-cold phosphate-buffered saline (PBS) before being scraped with a rubber policeman in 75μl lysis buffer: 50 mM Tris-HCl, pH 7.5; 150 mM NaCl; 1% triton X-100; 100 mM NaF; 2 mM Na_3_VO_4_; 20 mM Na_4_P_2_O_7_; 1 mM PMSF; protease inhibitor cocktail (Roche Diagnostics GmbH, Mannheim, Germany). Lysis was continued on ice for 30 min and insoluble components were subsequently pelleted by centrifugation for 25 min at 14.000 rpm and 4°C. Supernatants were stored at -20°C until further use. Protein concentrations were determined spectrophotometrically [33].

Protein samples (8 μg) were subjected to SDS-PAGE on 10% PA gels and blotted onto polyvinylidene fluoride (PVDF) membranes. Blots were blocked for 30 min using 3% non-fat dry milk in TBS-T (10 mM Tris-HCl, pH 8.0; 150 mM NaCl; 0.05% Tween-20) and incubated overnight at 4°C with a 1:2000 dilution of a phospho-ERK1/2 monoclonal antibody (#9106, Cell Signaling Technology, Beverly, USA). Following three washes with TBS-T, blots were incubated for 1 h with a 1:10,000 dilution of peroxidase-conjugated goat anti-mouse IgG (Pierce Biotechnology Inc., Rockford, IL, USA) in 3% non-fat dry milk in TBS-T. Immunoreactive bands were detected with Lumi-LightPLUS Western Blotting Substrate (Roche Diagnostics GmbH) using a BioChemi imaging system and obtained signals were quantified with the Labworks 4.0 program (UVP BioImaging Systems, Cambridge, UK). Blots were then stripped for 40 min at 60°C (in 62.5 mM Tris-HCl, pH 6.6; 100 mM β-mercapto-ethanol; 2% SDS), washed with TBS-T and reprobed for 1 h with rabbit polyclonal ERK1 (C-16) antibody (sc-93, Santa Cruz Biotechnology, Inc., Santa Cruz, USA) at a dilution of 1:8000 in TBS-T containing 3% non-fat dry milk. Peroxidase-conjugated goat anti-rabbit IgG (Pierce Biotechnology Inc.) was used for detection and quantitation as described above. Ratios of the phospho-ERK1/2/total ERK1 signals were determined for every sample and, to be able to compare signal ratios originating from different blots, a reference standard sample (corresponding to a PC12 cell lysate following 5 min treatment with EGF) was included on each blot.

PTPRR expression levels were determined by subjecting 50 μg of total protein to SDS-PAGE and immunoblot analysis as described above, using monoclonal antibody 6A6 [20]. Phospho-PTPPBSγ and phospho-PTPBR7 levels were determined by western blotting of 6A6 immunoprecipitates, performed as described previously [20], from PTPPBSγ or PTPBR7 expressing cells which had been incubated with 10 ng/ml EGF, 50 ng/ml NGF or 10 μM forskolin after overnight serum starvation. Blots were incubated simultaneously with a rabbit monoclonal phospho-PKA substrate (RRXS*/T*)(100G7) antibody (#9624, Cell Signaling Technology) and with 6A6, both in a 1:1000 dilution. Goat anti-mouse and goat anti-rabbit secondary antibodies conjugated to Alexa Fluor 680 (Molecular Probes) or IRDye800 (Rockland Immunochemicals) fluorescent dyes were used for detection and quantification on an Odyssey infrared imaging system (LI-COR).

### Immunofluorescence microscopy

Cells, cultured on collagen-coated coverslips, were fixed at room temperature for 30 min in PHEM buffer (60 mM PIPES; 25 mM HEPES, pH 6.9; 10 mM EGTA; 2 mM MgCl_2_) containing 4% paraformaldehyde, and subsequently washed with PBS and permeabilized for 5 min using 0.2% Triton X-100 in PBS. Aspecific sites were blocked by incubating for 30 min with 4% BSA in PBS before cells were incubated for 1 h with a 1:500 dilution of rabbit polyclonal α-SL antiserum [24] in PBS containing 0.4% BSA. Following three washes with PBS, cells were incubated for 1 h in a 1:300 dilution of Alexa568-conjugated goat-anti-rabbit IgG (Molecular Probes) in PBS, 0.4% BSA. Following PBS washes and methanol dehydration, coverslips were mounted on glass slides using Mowiol (Sigma-Aldrich), and images were collected using confocal laser scanning microscopy (MRC1024, Bio-rad).

## List of abbreviations

DUSP: dual-specificity phosphatase; EGF: epidermal growth factor; ERK: extracellular signal-regulated kinase; KIM: kinase interaction motif; MAPK: mitogen-activated protein kinase; MKP: MAP kinase phosphatase; NGF: nerve growth factor; PTP: protein tyrosine phosphatase; STEP; striatal-enriched phosphatase

## Declaration of competing interests

The author(s) declare that they have no competing interests.

## Authors' contributions

YN carried out the experiments and helped to draft the manuscript. PJ assisted in RNAi experiments. WH conceived and coordinated the study and drafted the manuscript. All authors read and approved the final manuscript.

## References

[B1] Chang L, Karin M (2001). Mammalian MAP kinase signalling cascades. Nature.

[B2] Marshall CJ (1995). Specificity of receptor tyrosine kinase signaling: transient versus sustained extracellular signal-regulated kinase activation. Cell.

[B3] Vaudry D, Stork PJ, Lazarovici P, Eiden LE (2002). Signaling pathways for PC12 cell differentiation: making the right connections. Science.

[B4] Pearson G, Robinson F, Beers Gibson T, Xu BE, Karandikar M, Berman K, Cobb MH (2001). Mitogen-activated protein (MAP) kinase pathways: regulation and physiological functions. Endocr Rev.

[B5] Yao Z, Seger R (2004). The molecular mechanism of MAPK/ERK inactivation. Curr Genomics.

[B6] Pulido R, Zúñiga A, Ullrich A (1998). PTP-SL and STEP protein tyrosine phosphatases regulate the activation of the extracellular signal-regulated kinases ERK1 and ERK2 by association through a kinase interaction motif. Embo J.

[B7] Muda M, Theodosiou A, Gillieron C, Smith A, Chabert C, Camps M, Boschert U, Rodrigues N, Davies K, Ashworth A, Arkinstall S (1998). The mitogen-activated protein kinase phosphatase-3 N-terminal noncatalytic region is responsible for tight substrate binding and enzymatic specificity. J Biol Chem.

[B8] Xu B, Stippec S, Robinson FL, Cobb MH (2001). Hydrophobic as well as charged residues in both MEK1 and ERK2 are important for their proper docking. J Biol Chem.

[B9] Blanco-Aparicio C, Torres J, Pulido R (1999). A novel regulatory mechanism of MAP kinases activation and nuclear translocation mediated by PKA and the PTP-SL tyrosine phosphatase. J Cell Biol.

[B10] Saxena M, Williams S, Tasken K, Mustelin T (1999). Crosstalk between cAMP-dependent kinase and MAP kinase through a protein tyrosine phosphatase. Nat Cell Biol.

[B11] Gronda M, Arab S, Iafrate B, Suzuki H, Zanke BW (2001). Hematopoietic protein tyrosine phosphatase suppresses extracellular stimulus-regulated kinase activation. Mol Cell Biol.

[B12] Braithwaite SP, Paul S, Nairn AC, Lombroso PJ (2006). Synaptic plasticity: one STEP at a time. Trends Neurosci.

[B13] Zúñiga A, Torres J, Úbeda J, Pulido R (1999). Interaction of mitogen-activated protein kinases with the kinase interaction motif of the tyrosine phosphatase PTP-SL provides substrate specificity and retains ERK2 in the cytoplasm. J Biol Chem.

[B14] Sharma E, Lombroso PJ (1995). A neuronal protein tyrosine phosphatase induced by nerve growth factor. J Biol Chem.

[B15] Zanke B, Suzuki H, Kishihara K, Mizzen L, Minden M, Pawson A, Mak TW (1992). Cloning and expression of an inducible lymphoid-specific, protein tyrosine phosphatase (HePTPase). Eur J Immunol.

[B16] Okamura A, Goto S, Nishi T, Hamasaki T, Ushio Y (1999). Overexpression of striatal enriched phosphatase (STEP) promotes the neurite outgrowth induced by a cAMP analogue in PC12 cells. Brain Res Mol Brain Res.

[B17] Shiozuka K, Watanabe Y, Ikeda T, Hashimoto S, Kawashima H (1995). Cloning and expression of PCPTP1 encoding protein tyrosine phosphatase. Gene.

[B18] Watanabe Y, Shiozuka K, Ikeda T, Hoshi N, Hiraki H, Suzuki T, Hashimoto S, Kawashima H (1998). Cloning of PCPTP1-Ce encoding protein tyrosine phosphatase from the rat cerebellum and its restricted expression in Purkinje cells. Brain Res Mol Brain Res.

[B19] Ogata M, Oh-hora M, Kosugi A, Hamaoka T (1999). Inactivation of mitogen-activated protein kinases by a mammalian tyrosine-specific phosphatase, PTPBR7. Biochem Biophys Res Commun.

[B20] Chirivi RG, Dilaver G, van de Vorstenbosch R, Wanschers B, Schepens J, Croes H, Fransen J, Hendriks W (2004). Characterization of multiple transcripts and isoforms derived from the mouse protein tyrosine phosphatase gene Ptprr. Genes Cells.

[B21] Brummelkamp TR, Bernards R, Agami R (2002). A system for stable expression of short interfering RNAs in mammalian cells. Science.

[B22] Yao H, York RD, Misra-Press A, Carr DW, Stork PJ (1998). The cyclic adenosine monophosphate-dependent protein kinase (PKA) is required for the sustained activation of mitogen-activated kinases and gene expression by nerve growth factor. J Biol Chem.

[B23] Dilaver G, Schepens J, van den Maagdenberg A, Wijers M, Pepers B, Fransen J, Hendriks W (2003). Colocalisation of the protein tyrosine phosphatases PTP-SL and PTPBR7 with beta4-adaptin in neuronal cells. Histochem Cell Biol.

[B24] van den Maagdenberg AM, Bächner D, Schepens JT, Peters W, Fransen JA, Wieringa B, Hendriks WJ (1999). The mouse Ptprr gene encodes two protein tyrosine phosphatases, PTP-SL and PTPBR7, that display distinct patterns of expression during neural development. Eur J Neurosci.

[B25] Blanchetot C, Chagnon M, Dube N, Halle M, Tremblay ML (2005). Substrate-trapping techniques in the identification of cellular PTP targets. Methods.

[B26] Mochizuki N, Yamashita S, Kurokawa K, Ohba Y, Nagai T, Miyawaki A, Matsuda M (2001). Spatio-temporal images of growth-factor-induced activation of Ras and Rap1. Nature.

[B27] Buschbeck M, Eickhoff J, Sommer MN, Ullrich A (2002). Phosphotyrosine-specific phosphatase PTP-SL regulates the ERK5 signaling pathway. J Biol Chem.

[B28] Rintelen F, Hafen E, Nairz K (2003). The Drosophila dual-specificity ERK phosphatase DMKP3 cooperates with the ERK tyrosine phosphatase PTP-ER. Development.

[B29] Muñoz JJ, Tárrega C, Blanco-Aparicio C, Pulido R (2003). Differential interaction of the tyrosine phosphatases PTP-SL, STEP and HePTP with the mitogen-activated protein kinases ERK1/2 and p38alpha is determined by a kinase specificity sequence and influenced by reducing agents. Biochem J.

[B30] Augustine KA, Silbiger SM, Bucay N, Ulias L, Boynton A, Trebasky LD, Medlock ES (2000). Protein tyrosine phosphatase (PC12, Br7,S1) family: expression characterization in the adult human and mouse. Anat Rec.

[B31] Hendriks W, Schepens J, Bächner D, Rijss J, Zeeuwen P, Zechner U, Hameister H, Wieringa B (1995). Molecular cloning of a mouse epithelial protein-tyrosine phosphatase with similarities to submembranous proteins. J Cell Biochem.

[B32] Kinsella TM, Nolan GP (1996). Episomal vectors rapidly and stably produce high-titer recombinant retrovirus. Hum Gene Ther.

[B33] Bradford MM (1976). A rapid and sensitive method for the quantitation of microgram quantities of protein utilizing the principle of protein-dye binding. Anal Biochem.

